# Feeding Sugar to Tumors: With a Supplement of miR?

**DOI:** 10.1161/JAHA.112.006213

**Published:** 2012-12-19

**Authors:** Olivier P. Blanc‐Brude, Alain Tedgui

**Affiliations:** 1 INSERM UMR‐S 970, Paris Cardiovascular Research Center – PARCC, Université Paris Descartes, Sorbonne Paris Cité, Paris, France (O.P.B.B., A.T.)

**Keywords:** editorials, angiogenesis, glucose, tumor

## Introduction

Thrombospondin‐1 (TSP‐1) is a 450‐kDa adhesive glycoprotein that was initially discovered in platelets and subsequently in a variety of cell types. TSP‐1 is one of the first physiological inhibitors of angiogenesis to be identified and remains one of the most potent and best described.^1^ TSP‐1 is a sophisticated multidomain regulator of tissue angiogenesis that stimulates intense cytoskeletal remodeling and cell surface protein activation in multiple cell types. Prolonged exposition of endothelial cells to TSP‐1 is followed by apoptosis, and TSP‐1 was thus deemed an antiangiogenic factor. More recently, Isenberg, Robert, and colleagues^2–3^ demonstrated that TSP‐1 limits tissue perfusion by targeting vascular function as well as vascular geometry through specific inhibition of nitric oxide signaling. TSP‐1 can thus counterbalance the effects of proangiogenic factors like VEGF and nitric oxide in several ways and reverse proangiogenic equilibrium. It has been suggested that TSP‐1 overexpression is deleterious in the largely ischemic context of diabetes. And vvTSP‐1 may also play a part in tumor growth and vascularization.

## Integration of miR Repression of TSP‐1 With Other Regulation Mechanisms

Bhattacharyya and colleagues^4^ previously described that high glucose induces TSP‐1 expression in macro‐ and microvascular endothelial cells of diverse tissue origin. Glucose is thought to upregulate TSP‐1 gene (*THBS1*) transcription in endothelial cells through activation of the transcription factor aryl hydrocarbon receptor,^5^ which can form complexes with egr‐1 and activator protein‐2, 2 transcription factors that are also upregulated in diabetes. Others have shown that glucose also upregulates THBS‐1 expression in vascular smooth muscle and mesangial cells.^6–7^ TSP‐1 mRNA is overexpressed in diabetic bone marrow and endothelial progenitor cells and may even remain upregulated long after cell transplantation, with a functional antiangiogenic impact.^8^ It is thus clear that high glucose stimulates THBS‐1 expression, and the posttranscriptional regulation of TSP‐1 may be a critical last barrier before deleterious TSP‐1 expression in diabetic blood vessels.

TSP‐1 production is regulated by microRNA (miRNA) in tumors. miR has the ability to bind and sequester RNA in a pool that is devoid of polysomes so that they are not actively translated. Hence, miR can suppress protein production despite transcriptional upregulation. Bhattacharyya and colleagues previously suggested that the 3′‐UTR of TSP‐1 mRNA plays a critical role in the silencing of TSP‐1 expression in microvascular endothelial cells.^4^ Furthermore, high‐glucose‐induced posttranscriptional regulation of TSP‐1 may even be responsible for cell‐type‐specific regulation of TSP‐1 protein expression. In this issue of *JAHA*, Bhattacharyya and colleagues^9^ complete their description of this molecular machinery and identify miR‐467 as a critical translational suppressor of TSP‐1 mRNA via direct binding to the 3′‐UTR. This makes miR‐467 a new critical physiological inhibitor of TSP‐1 and facilitator of angiogenesis.

## Relevance for Diabetes

In this study, 2 mouse models of diabetes (leptin receptor knockout and streptozotocine‐induced pancreatic dysfunction) helped to show that hyperglycemia promotes miR‐467 upregulation, TSP‐1 repression, and angiogenesis. Bhattacharyya and colleagues report widespread effects of hyperglycemia on miR‐467 upregulation in the heart, lungs, and kidneys, and more particularly in endothelial cells. However, diabetes is associated with degenerative complications and hypoxic events in these organs, with endothelial dysfunction, vascular wall thickening, and progressive sclerosis in connection with TSP‐1 upregulation. Indeed, TSP‐1 is increased in diabetic patient plasma^10^ and in critically ischemic diabetic limbs.^11^ TSP‐1 gene and protein expression are increased by low nitric oxide in endothelial cells and hypoxia in ischemic tissues, potentially through the posttranslational stabilization of TSP‐1 mRNA.^12^ It is thus not straightforward to reconcile the new miR‐467 upregulation and putative angiogenesis with diabetic vascular lesions or tissue ischemia. Bhattacharyya and colleagues^4^ suggest that an explanation may lie in cell‐type‐specific responses to high glucose, leading to raised TSP‐1 protein expression in large vessels^13^ but repression in microvascular endothelial cells. Further studies will be needed to fully understand the clinical significance of these observations. We now need to understand the tissue‐specific regulation of miR‐467 and how its differential expression may regulate the perfusion of different diabetic tissues. Indeed, why does the high‐glucose‐stimulated miR‐467 pathway not operate to block TSP‐1 overexpression in the long term or to maintain the diabetic vascular network? Alternatively, if the miR‐467 pathway only operates in microvessels, how can perfused and growing tumors not be subjected to the antiangiogenic effects of circulating TSP‐1?

One straightforward implication of miR‐467 upregulation by high glucose relates to diabetic retinopathy. TSP‐1 induction plays a key role in modulating the response to retinal hypoxia,^14^ but TSP‐1 was reported as undetectable in the vitreous fluid of patients with proliferative diabetic retinopathy and active neovascularization,^15^ suggesting specific downregulation in the eye.^16^ Bhattacharyya had previously reported the posttranscriptional repression of TSP‐1 in different retina cells.^4^ Another application of the finding may relate to diabetic nephropathy, where TSP‐1 overexpression is thought to contribute to tubule hypertrophy and sclerosis in connection with TSP‐1‐mediated TGF‐β1 activation. MiR‐467 manipulation may thus represent a promising therapeutic avenue for these syndromes.

## Relevance for Cancer

**Figure 1.**
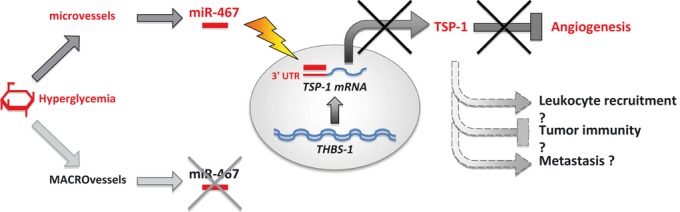
Impact of hyperglycemia on endothelial and carcinoma tumor cells. Hyperglycemia stimulates the expression miR‐467 in microvascular and carcinoma cells, and miR‐467 can then repress TSP‐1 mRNA translation. Hence, miR‐467 expression can lift the TSP‐1‐mediated inhibition of angiogenesis in the solid tumor environment, enabling excessive angiogenesis (TSP‐1, thrombospondin‐1). Given the previously reported roles of TSP‐1 in leukocyte recruitment, immune cell survival and activation, and tumor metastasis, one may wonder what impact miR‐467 manipulation may have in the general context of cancer growth and dissemination, beyond murine models of hyperglycemia.

Here, Bhattacharyya and colleagues suggest that miR‐467‐mediated repression of TSP‐1 may be particularly relevant to tumor growth in the presence of hyperglycemia. Their mouse models mimicked diabetes in association with the subcutaneous injection of prostate and mammary carcinoma cells and tumor growth monitoring. These elegant models are convenient to demonstrate the impact of hyperglycemia on TSP‐1 expression and solid tumor early implantation. But TSP‐1 is also recognized as promoting tumor cell migration, adhesion, and metastatic dissemination.^17^ MiR‐467 expression and TSP‐1 repression might thus favor the growth of the tumor while reducing metastasis. When therapeutic intervention with antagomiR‐467 is tested again in a model of cancer growth, it would be interesting to assess whether restored TSP‐1 expression triggers metastasis. Further work will be necessary to fully understand the clinical significance of TSP‐1 repression in cancer (Figure 1).

## Tissue Neovascularization and Vascular Remodeling

TSP‐1 is thought to play an important part in the regression of nonperfused or abnormal microvessels, the adaptation of diameter to blood flow, and the optimization of the vascular tree after neovascularization.^18^ A recent body of work demonstrated that TSP‐1 targets vascular function and limits endothelial‐mediated vasodilation by inhibiting nitric oxide signaling in vascular smooth muscle.^2–3^ In the discussed study, vascular exploration was limited to the quantification of total cell infiltration and CD31+ vascular structures in tumors as indices of angiogenesis. In light of the previous reports, one may wonder if the new vessels formed during hyperglycemia are functional and perfused. What is the impact of miR‐467 on vasodilation and permeability? The repression of TSP‐1 by miR‐467 in solid malignant tumors might help to promote the maintenance of overly complex or dysfunctional capillary networks unrelated to tissue perfusion or oxygenation. The same questions may apply to antagomiR‐467‐treated tissues.

## Tissue Remodeling Beyond the Blood Vessel

In the wider picture, the model of solid tumor growth can be envisaged as a model of hyperglycemia‐induced tissue remodeling. The description of miR‐467 repression of TSP‐1 may prove relevant to other inflammation‐mediated tissue remodeling processes and prompts questions related to the multiple functions of TSP‐1. For instance, TSP‐1 is known as a potent stimulus of cell‐surface integrin activation, circulating cell adhesion, and leukocyte transendothelial migration, participating in tissue inflammation. TSP‐1 deficiency was recently shown to reduce obesity‐associated inflammation^19^ and triggers specific responses related to macrophage activation and phagocytosis. TSP‐1 promotes macrophage infiltration of the M1 subtype, contributing to antimelanoma immunity^20^ and ischemic inflammation.^21^ On certain leukocyte subtypes, like lymphocytes, prolonged contact of TSP‐1 is known to trigger apoptosis. In addition, TSP‐1 modulates TGF‐β1 activation in smooth muscle cells, fibroblasts, and mesangial cells and mediates the synthesis and extracellular deposition of collagens and fibronectin.^22^ This TSP‐1‐mediated pathway is stimulated by hyperglycemia and comes into play during diabetic nephropathy.^23^ One could thus imagine that miR‐467 expression and TSP‐1 repression during hyperglycemia induce a thorough transformation of the inflammatory infiltrate profile and reduce connective tissue deposition. In solid tumors, the modified immunoinflammatory response may affect tumor angiogenesis and perfusion beyond the direct effects of TSP‐1 on endothelial biology. These aspects can be anticipated to be critical for tumor growth.

In summary, the study by Bhattacharyya identifies miR‐467 as a new therapeutic opportunity to modulate angiogenesis by lifting or enforcing TSP‐1‐mediated checkpoints. The study also raises novel possibilities for investigating the “sweet and sour” roles of TSP‐1 at the crossroad between inflammation, tissue remodeling, and the immune response.
